# Glycotyping of *Trypanosoma brucei* variant surface glycoprotein MITat1.8

**DOI:** 10.1016/j.molbiopara.2010.06.007

**Published:** 2010-11

**Authors:** Angela Mehlert, Lauren Sullivan, Michael A.J. Ferguson

**Affiliations:** Division of Biological Chemistry and Drug Discovery, College of Life Sciences, University of Dundee, Dundee DD1 5EH, United Kingdom

**Keywords:** *Trypanosoma brucei*, N-linked oligosaccharides, N-glycosylation, Glycosylphosphatidylinositol, GPI, Mass spectrometry

## Abstract

Following a switch from variant surface glycoprotein MITat1.4 to variant surface glycoprotein MITat1.8 expression by Lister strain 427 *Trypanosoma brucei brucei* parasites, the latter uncharacterized variant surface glycoprotein was analysed. Variant surface glycoprotein MITat1.8 was found to be a disulphide-linked homodimer, containing a complex N-linked glycan at Asn58 and a glycosylphosphatidylinositol membrane anchor attached to Asp419. Mass spectrometric analyses demonstrated that the N-glycan is exclusively Galβ1-4GlcNAcβ1-2Manα1-3(Galβ1-4GlcNAcβ1-2Manα1-6)Manβ1-4GlcNAcβ1-4GlcNAc and that the conserved Man_3_GlcN-*myo*-inositol glycosylphosphatidylinositol anchor glycan core is substituted with an average of 4 hexose, most likely galactose, residues. The presence of a complex N-glycan at Asn58 is consistent with the relatively acidic environment of the Asn58 N-glycosylation sequon, that predicts N-glycosylation by *T. brucei* oligosaccharyltransferase TbSTT3A with a Man_5_GlcNAc_2_ structure destined for processing to a paucimannose and/or complex N-glycan (Izquierdo L, Schulz B, Rodrigues JA et al. *EMBO J* 2009;28:2650–61 [12]).

## Graphical abstract

VSG MITat1.8 was characterized with respect to its N-glycosylation, GPI anchor structure and found to be a disulfide-linked homodimer.

The tsetse transmitted African trypanosomes of the *Trypanosoma brucei* group are the causative agents of Human African Trypanosomiasis, also known as African sleeping sickness, and nagana in cattle. Trypanosomiasis is a major human and veterinary problem in 36 countries of sub-Saharan Africa. *T. brucei* is able to evade its hosts’ immune system by antigenic variation, whereby the major coat protein, the variant surface glycoprotein (VSG), may be switched for a different variant from a repertoire of many distinct VSG genes [1]. However, individual trypanosomes only express a single VSG gene at a time through allelic exclusion [2].

Laboratory research on trypanosome biochemistry tends to use clones of Lister strain 427 *T. brucei* that express single VSG genes and VSG proteins. Normally, in acute (three-day) animal infections, the trypanosome population remains clonal with respect to VSG expression. However, from time to time, antigenic variation to another VSG variant may result in a population of trypanosomes in an animal infection expressing both the parental and a new, switched, VSG variant. We observed such a switch using a stabilate expressing VSG variant MITat1.4 (also known as VSG117 and Lister 427-4) that had been expanded in animals and re-stabilated twice before. The following describes how we identified and characterized the switched VSG variant, including its post-translational modifications.

Following the infection of mice or rats with a *T. brucei* stabilate previously thought to be clonal for VSG MITat1.4 expression, soluble form VSG (sVSG) was purified from the parasites using a method that allows the *T. brucei* GPI-specific phospholipase (GPI-PLC) to cleave the *sn*-1,2-dimyristoylglycerol lipid portion of its GPI anchor [3,4]. The presence of two different VSGs was apparent by reducing SDS-PAGE and Safely Blue (Sigma) staining (Fig. 1A, lane 1). A similar sample was dissolved in SDS sample buffer and subjected to reduction and alkylation with iodoacetamide prior to SDS-PAGE on a precast SDS-PAGE gradient gel (4–12% bis–tris gel from Invitrogen). After staining, the two VSG bands were excised and processed for in-gel tryptic digestion [5]. The extracted peptides were analyzed by LC–MS/MS by the University of Dundee Fingerprints Proteomics Facility using a Thermo Orbitrap XL instrument and the results were analyzed using Mascot (Matrix Science). The upper VSG band was identified as VSG MITat1.4 (73% sequence coverage, Mascot score 5054) and the lower as VSG MITat1.8 (66% sequence coverage, Mascot score 4341). The expression of VSG MITat1.8 (also known as variant Lister 427-8) has been previously described, at least at the mRNA level, during a VSG switch from VSG MITat1.5 (also known as variant 118 and Lister 427-5) expression [6]. The full DNA sequence for the VSG MITat1.8 gene was subsequently deposited by Cross and Dreesen (accession number AAX36024). Consistent with the proteomic identification of two VSGs purified from the trypanosome population, immunofluorescence microscopy of fixed trypanosomes using rabbit anti-VSG MITat1.4 serum and FITC-labelled mouse anti-rabbit IgG showed that only about half of the trypanosomes expressed cell surface VSG MITat1.4 (data not shown). VSG MITat1.8 can be classified as an A1 VSG, based on N- and C-terminal Cys residue patterns, using the nomenclature of Carrington et al. [7].

When a sample of the mixed sVSGs was analysed by MALDI-Tof mass spectrometry, it appeared that, whereas sVSG MITat1.4 was observed as a set of overlapping monomeric glycoforms centred around 53.3 kDa, sVSG MITat1.8 appeared mostly in its dimeric form at approximately 96.5 kDa although a monomeric peak was also seen at approximately 48.3 kDa (data not shown). Although most known VSGs are non-covalent homodimers, [8,9], one *T. evansi* VSG [10] and one *T. brucei* VSG (ANTat1.1) have been described as a predominantly disulphide-linked homodimers [11]. We therefore analyzed the mixed sVSG preparation by SDS-PAGE without reduction (Fig. 1A, lane 2). In this case, the two sVSG bands separated dramatically, with one running with the apparent molecular weight of a monomer and the other as a dimer. Excision of the bands for tryptic digestion and proteomic analyses revealed that the monomeric sVSG was MITat1.4 whereas the dimeric sVSG was MITat1.8. From these data, we can conclude that VSG MITat1.8 is predominantly an inter-chain disulphide-linked homodimeric VSG.

We were encouraged to study VSG MITat1.8 further for the following reasons. Firstly, there are relatively few well-characterized VSGs with respect to their post-translational modifications. Secondly, following our recent work analyzing the *T. brucei* bloodstream form N-glycosylation site occupancies [12], we were interested to use it as a test-case to see if we could predict the N-glycan type(s) (oligomannose or paucimannose/complex) that it should contain. Thirdly, we were interested to see whether the extent of GPI anchor sidechain galactosylation would be similar to those of other VSGs with type-1 C-terminal domains.

The predicted amino acid sequence of VSG MITat1.8 suggests a cleavable N-terminal signal peptide (residues 1–24) [13] and a C-terminal GPI anchor signal peptide (residues 420–442) [14]. In the predicted mature protein sequence, the C-terminal residue (Asp419) is attached to a GPI membrane anchor and there is a single potential N-linked glycosylation sequon (NXS/T, X ≠ P) at Asn58 within the sequence TEGLL**NAT**DEIAL. This relatively acidic sequence (*i.e.,* the glycosylation sequon ± 5 amino acid residues) has a pI of 3.57, which according to previous work in our laboratory [12], suggests that it has a high probability (>85%) of being modified exclusively by the TbSTT3A oligosaccharyltransferase leading to occupancy by paucimannose and/or complex, rather than oligomannose, N-glycans. With respect to the GPI anchor sidechains, the type-1 C-terminal domain structure of VSG MITat1.8 would suggest that the GPI anchor is modified with galactose-containing sidechains with a mean of about 3 Gal residue per anchor, as opposed to about 5 and 0 Gal residues per anchor for type-2 and type-3 C-terminal domain VSGs, respectively [15–20].

Paucimannose and complex N-glycans can be discriminated from oligomannose N-glycans on the basis of their resistance and sensitivity, respectively, to the enzyme endoglycosidase H (Endo H) [21], while both are sensitive to peptide N-glycosidase F (PNGase F). The mixed VSG preparation was therefore digested with each enzyme [12] (Fig. 1B, lanes 1–3), alongside control digests with pure sVSG MITat1.4 (Fig. 1B, lanes 4–6). Digestion with PNGase F caused a similar reduction in apparent molecular weight for both sVSGs (Fig. 1B, lane 3) whereas digestion with Endo H caused a reduction in apparent molecular weight of only the upper sVSG (MITat1.4) band, which merged with the lower sVSG MITat1.8 band (Fig. 1B, lane 1). The sensitivity of the MITat1.4 band to Endo H is consistent with the known occupancy of its single N-glycosylation site at Asn 420 by a Man_9_GlcNAc_2_ to Man_6_GlcNAc_2_ oligomannose series [22]. In contrast, the current data are consistent with sVSG MITat1.8 containing a single Endo H-resistant (paucimannose/complex) N-glycan.

To further analyse the N-linked glycans of the mixed VSGs, an aliquot was digested with Pronase and the N-linked glycopeptides purified by QAE-Sephadex anion-exchange chromatography, as described in [16]. The N-linked glycopeptide fraction elutes relatively early in the ammonium acetate gradient (80 mM) and, after freeze-drying to remove the ammonium acetate, small aliquots were loaded into gold-coated glass nanospray tips (Waters Type-F) for analysis on an ABI Q-StarXL mass spectrometer. In the primary ES-MS spectrum (Fig. 2A), ion clusters of [M+2H]^2+^, [M+H+Na]^2+^ and [M+H+K]^2+^ corresponding to the oligosaccharides Man_9_GlcNAc_2_ to Man_6_GlcNAc_2_ linked to the peptide NST were observed, as expected for sVSG MITat1.4 [22]. However, in addition, a single ion cluster consistent with the [M+2H]^2+^, [M+H+Na]^2+^ and [M+H+K]^2+^ ions of composition Hex_5_HexNAc_4_ linked to the peptide NAT from sVSG MITat1.8 was observed. These assignments were confirmed by ES-MS/MS of the [M+2H]^2+^ ion of each cluster. The MS/MS spectrum of *m/z* 964.44 is shown in (Fig. 2B), illustrating how the NAT peptide component was deduced. The intense *m/z* 366 product ion indicates that the glycan has two non-reducing Hex-HexNAc termini. Taken together with GC–MS compositional analysis of the N-glycopeptide fraction (that showed the presence of only Man, Gal and GlcNAc) as well as precedent from other VSG N-glyan structures [23,24], we conclude that Asn58 of sVSG MITat1.8 is occupied exclusively by a simple neutral asiolo biantennary (NA2) complex glycan of Galβ1-4GlcNAcβ1-2Manα1-3(Galβ1-4GlcNAcβ1-2Manα1-6)Manβ1-4GlcNAcβ1-4GlcNAc.

We then analysed the GPI anchor glycan structures using an off-blot method that we have recently developed [18,25]. The chemical nature of GPI anchors, in which the glycans are joined to the C-terminal amino acid of the protein via an ethanolamine phosphate bridge and to the lipid portion of the anchor via another phosphate bond, permit the use of aq. HF digestion to remove the GPI glycan from the protein and lipid or, in this case, from the phosphate remnant of the lipid following GPI-PLC cleavage [16]. Thus, aq. HF digestion of regions of polyvinylidene fluoride (PVDF) Western blots carrying GPI-proteins allows the release the glycans while the protein portion remains on the membrane. A PVDF Western blot was prepared containing the mixed sVSG preparation and control samples of pure sVSG MITat1.4 (a type-1 VSG) and sVSG MITat1.2 (a type-2 VSG, also known as variant 221 and Lister 427-2) which have known GPI structures [16,18]. After staining the blot with amido black, the pieces of PVDF corresponding to the upper and lower sVSG bands of the mixed sVSG sample, and of the control sVSG bands, were excised and put in separate Eppendorf tubes. Each band was wetted with methanol and then treated with 40 μl aqueous HF for 60 h at 0 °C. The aq. HF was removed and retained and the strip washed with twice 100 μl water. The water washes and aq. HF were combined, lyophilized, redissolved in 50 μl water and transferred to a 2 ml reactivial (Pierce). Following drying and re-drying from 50 μl methanol in a Speedvac concentrator (Thermo), the GPI glycans were permethylated using the method described in [26]. The resulting permethylated glycans, which each carry a positively charged quaternary ammonium group from the permethylation of the GPI glycan glucosamine residue, were dried and dissolved in a small volume of methanol. An aliquot (1 μl) mixed with the same volume of 2,5-dihydroxybenzoic acid matrix and loaded onto a MALDI plate. The glycans were analysed in positive ion mode using an ABI Voyager DE-STR MALDI-Tof mass spectrometer. The control sVSGs produced the expected profiles, with sVSG MITat1.2 containing an average of about 5 Gal sidechain residues (Fig. 2C) and sVSG MITat1.4 from the upper band and the control lane containing an average of about 3 Gal sidechain residues (Fig. 2D). The permethylated GPI glycans of sVSG MITat1.8 (Fig. 2E) are roughly intermediate in size, containing an average of about 4 hexose sidechain residues. We assume that the side chain hexoses are galactose as the only monosaccharides found in the GC–MS compositional analysis of the GPI fraction from the ion exchange column were galactose and mannose.

In summary, we may make the following conclusions from the data described in this brief report. Firstly, VSG MITat1.8 expressing cells presumably have a small growth advantage over VSG MITat1.4 expressing cells *in vivo*, otherwise this variant could not appear as a significant portion of the trypanosome population. Secondly, VSG MITat1.8 is the third example of a covalent, disulphide bonded, *T. brucei* VSG dimer. Based on known disulphide bonding patterns of VSGs, the most likely residue for inter-chain disulphide bonding is Cys336. Thirdly, as predicted from the relatively acidic environment around Asn58 of the VSG MITat1.8 glycosylation sequon [12], the VSG carries exclusively an NA2 complex biantennary N-glycan. The lack of glycan microheterogeneity at this N-glycosylation site is relatively unusual for glycoproteins in general and unique for a VSG. The NA2 structure, for example, is substituted with one or more Galβ1-4GlcNAc (LacNAc) repeats or with one or two αGal residues in 1–3 linkage in VSGs MITat1.7 and MITat1.5, respectively [23,27]. Finally, the level of GPI anchor substitution is intermediate between those described previously for type-1 VSGs and type-2 VSGs, suggesting that it may be wrong to predict GPI anchor substitution based on C-terminal domain type alone. The aforementioned structural features are built into a model for VSG MITat1.8 (Fig. 3).

## Figures and Tables

**Fig. 1:**
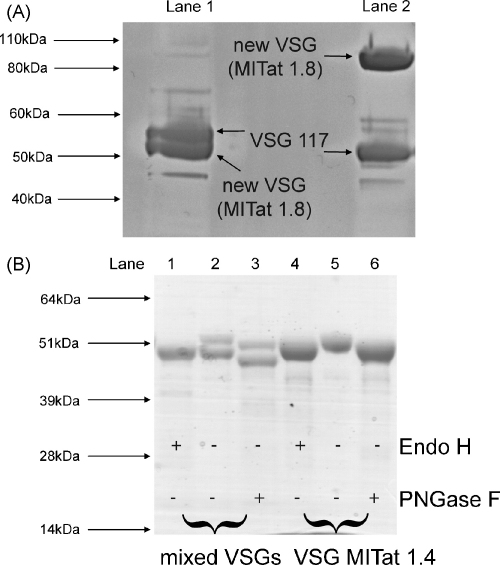
SDS-PAGE and Coomassie blue staining of sVSGs with and without reduction and before and after endoglycosidase digestion. (A) SDS-PAGE showing the unusual behaviour of MITat1.8 VSG. Lane 1 shows the mixture of VSGs (MITat1.4 and 1.8) with reducing sample buffer, lane 2 shows the same mixture with non-reducing sample buffer. The MITat1.8 VSG is seen electrophoresing as a dimer at an apparent molecular weight of over 80 kDa. (B) SDS-PAGE showing endoglycosidase sensitivities of the mixed sVSG MITat1.4 and 1.8 sample. Lanes 1–3 were loaded with the mixed of sVSG MITat1.4 and sVSG 1.8 VSGs and lanes 4–6 were loaded with pure sVSG MITat1.4. Lanes 2 and 5 show untreated control sVSGs, the EndoH treated samples are to the left of the control (lanes 1 and 4) and the PNGaseF treated ones to the right (lanes 3 and 6).

**Fig. 2:**
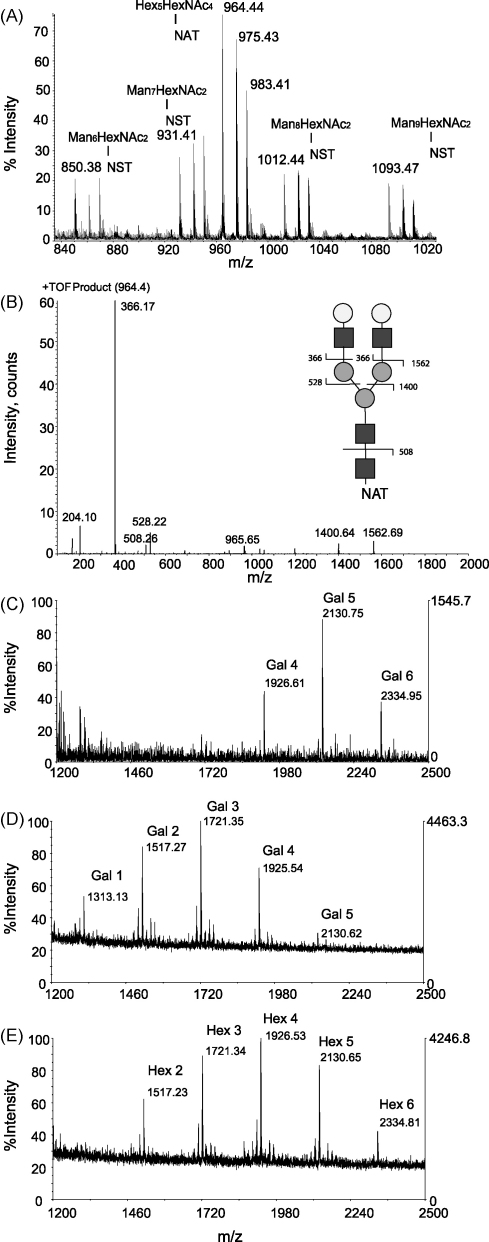
Mass spectrometric analyses of Pronase N-glycopeptides and permethylated GPI glycans. (A) Positive ion ES-MS spectrum of Pronase N-glycopeptides prepared from the mixture of sVSG MITat1.4 and sVSG MITat.8. The clusters of ions marked Man_6–9_GlcNAc_2_-NST correspond to the [M+2H]^2+^, [M+H+Na]^2+^ and [M+H+K]^2+^ of the expected N-glycopeptides from sVSG MITat1.4 [22]. The cluster of ions at *m/z* 964.44, 975.43 and 983.41 marked correspond to the [M+2H]^2+^, [M+H+Na]^2+^ and [M+H+K]^2+^ ions of composition Hex_5_HexNAc_4_-NAT from sVSG MITat1.8. (B) Product ion spectrum of the 964.44 ion from A showing a pattern that is distinctive for the NA2 N-linked oligosaccharide attached to the peptide NAT, see inset structure for the assignments of the product ions. Dark grey squares = N-acetylglucosamine (GlcNAc), light grey circles = mannose (Man) and open circles = galactose (Gal). (C–E) Positive ion MALDI-Tof mass spectra of permethylated GPI glycans from (C) sVSG MITat1.2, (D) sVSG MITat1.4 (upper band from Fig. 1B) and (E) sVSG MITat1.8 (lower band from Fig. 1B). The annotated ions correspond to the permethylated GPI glycan core of Man_3_GlcNMe_3_-inositol with 2–6 side chain Gal [16,18] or Hex (hexose) residues, as indicated.

**Fig. 3:**
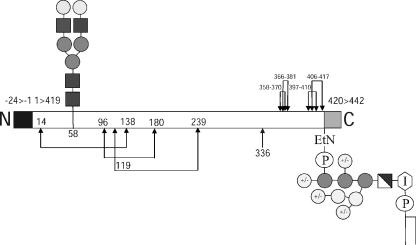
Diagrammatic representation of VSG MITat1.8. The cleaved N-terminal signal sequence (amino acid residues −24 to −1) is represented by the black box, the cleaved C-terminal GPI signaling sequence (amino acid residues 420–442) by the grey box. The linked arrows represent the predicted disulphide bonding pattern of the protein based on previously reported data and the single arrow at 336 is the cysteine residue through which inter-chain disulphide binding is most likely to occur. The N-link oligosaccharide at N58 is of the NA2 type structure, the GPI anchor is intermediate in average size to those of VSGs MITat1.4 and MITat1.2. In this model, we are assuming that the additional hexose residues observed in Fig. 2E are galactose residues linked to the conserved Man_3_GlcN-*myo*-inositol. Dark grey and white square = glucosamine (GlcN), light grey circles = mannose (Man), open circles = galactose (Gal), symbol at far right represents *sn*-1,2-dimyristoyl-phosphatidylinositol.
